# Large Colloidal InAs Nanocrystals Synthesized with a Grignard Reagent

**DOI:** 10.1021/acs.chemmater.6c00517

**Published:** 2026-06-12

**Authors:** Guncem Ozgun Eren, Houman Bahmani Jalali, Hossein Roshan, Mauro Garbarino, Yurii P. Ivanov, Mirko Prato, Giorgio Divitini, Luca De Trizio, Liberato Manna, Francesco Di Stasio

**Affiliations:** † Photonic Nanomaterials, 121451 Istituto Italiano di Tecnologia, Genova 16163, Italy; ‡ Nanochemistry, 121451 Istituto Italiano di Tecnologia, Genova 16163, Italy; § Dipartimento di Chimica e Chimica Industriale, Università̀ degli Studi di Genova, Genova 16146, Italy; ∥ Electron Spectroscopy and Nanoscopy, 121451 Istituto Italiano di Tecnologia, Genova 16163, Italy; ⊥ Materials Characterization, 121451 Istituto Italiano di Tecnologia, Genova 16163, Italy; # Chemistry Facility, 121451 Istituto Italiano di Tecnologia, Genova 16163, Italy

## Abstract

Colloidal nanocrystals (NCs) active in the near-infrared (NIR) region are appealing for a variety of applications, such as machine vision, hyperspectral imaging, and medical diagnosis. Among the various classes of NIR-active NCs, InAs stands out as one of the most viable compositions in terms of environmental safety. Yet, the synthesis of InAs NCs often relies on expensive and hazardous As precursors, such as tris­(trimethylsilyl)­arsine. Alternative syntheses have also been developed, mainly exploiting tris­(dimethylamino)-arsine (amino-As) in conjunction with a reducing agent (typically organometallic metal hydrides) as a safer and more economical precursor. However, to date, the colloidal synthesis of large InAs NCs (active in the 1000–1600 nm window) using the amino-As route relies on lengthy continuous-injection approaches. In the present study, we demonstrate that using benzyl magnesium chloride (a commercially available Grignard compound) as the reducing agent enables the synthesis of InAs NCs with optical absorption tunable from 835 nm (≈3 nm NC diameter) to 1180 nm (NC diameter of ≈4.2 nm) via a single hot injection procedure. After a ZnSe shell growth, the resulting InAs@ZnSe core@shell NCs exhibit photoluminescence tunable from 1000 to 1500 nm, with photoluminescence quantum yields of 46%, 16%, and 6% at 1030, 1190, and 1500 nm, respectively. We further demonstrate that the synthesis of InAs NCs is compatible with a variety of commercially available Grignard reagents.

## Graphical abstract

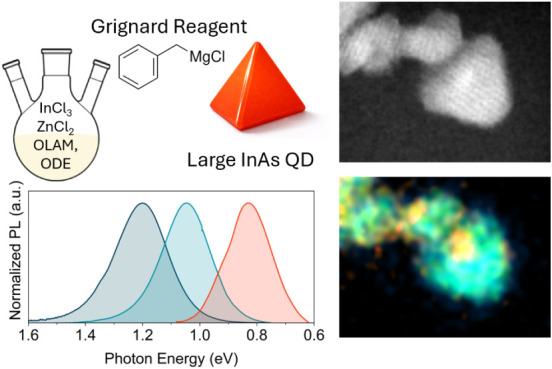

## Introduction

The interest in infrared (IR) emitting colloidal nanocrystals (NCs) has increased considerably in recent years, thanks to their appealing size-dependent optical and electronic properties and solution processability.^1,2^ Furthermore, the facile integration of IR NCs with complementary metal-oxide-semiconductor technologies could lead to low-cost optoelectronic devices such as light-emitting diodes,^3–6^ photovoltaics,^7^ sensors,^8^ solar cells,^7,9^ and lasers,^10,11^ which are employed in diverse applications (e.g., night vision,^12^ telecommunication,^3^ bioimaging,^13^ and security authentication^14^). The variety of suitable materials that can be used in the IR is limited compared to the visible spectral range.^2,4,15^ In this regard, the most developed IR-emitting NCs are lead (Pb)- or mercury (Hg)-based chalcogenides;^16,17^ however, they are not compliant with the Restriction of Hazardous Substances (RoHS) directive due to the toxicity of both Pb and Hg, posing a major obstacle to their implementation in commercial products.^18^

These limitations have promoted research and development of environmentally friendly IR-emitting materials, including Ag- and Cu-based I–III–VI semiconductors (namely, CuInSe_2_, CuInS_2_, AgInSe_2_),^19^ Ag chalcogenides (Ag_2_Te),^20^ and In-based III–V semiconductors such as InSb and InAs.^21–23^ Among these, InAs NCs stand out as promising candidates for IR optoelectronic applications thanks to their narrow bulk bandgap of 0.35 eV^2^ and large exciton Bohr radius (reported values ranging from ≈45 nm^24^ to ≈31.2 nm^25^), resulting in an optical absorption (Abs) peak tunable from ≈700 nm up to ≈2600 nm.^26–30^ To date, the synthesis of InAs NCs has been carried out by employing various As precursors^31–33^ with tris­(trimethylsilyl)­arsine (TMS-As) being the most commonly used one to prepare NCs with high control over their size distribution, leading to sharp optical Abs peaks (<50 meV half-width at half-maximum, HWHM).^26,29,34^ However, the shortcomings of this precursor include pyrophoricity, inherent toxicity, high cost, and limited commercial availability. These limitations have prompted various groups to utilize alternative As sources for the synthesis of InAs NCs,^35–40^ among which tris­(dimethylamino)­arsine (amino-As) has emerged thanks to its safe handling, commercial availability, and low price.^28,35,41^

For the synthesis of InAs NCs via the amino-As route, the precursor must be used together with a reducing agent to reduce As (+III) to As (−III). It has been demonstrated that the type of reducing agent plays an important role in governing the kinetics of nucleation and growth of the resulting InAs NCs.^22,30,42–44^ In this regard, various types of reducing agents have been tested by different research groups: lithium bis­(trimethylsilyl)­amide, 1,1,3,3,5,5-hexamethyltrisiloxane, diisobutylaluminum hydride (DIBAL-H), alane *N,N*-dimethylethylamine (DMEA-AlH_3_), and trioctylamine-alane (TOA-AlH_3_).^28,30,43,44^ Among them, DMEA-AlH_3_ and TOA-AlH_3_ are regarded as the most effective reducing agents, in terms of good control over the size distribution of InAs NCs.^38,43,45^ Moreover, Zhu et al. demonstrated that if amino-As-based InAs NCs are synthesized with DMEA-AlH_3_ as the reducing agent and ZnCl_2_ as an additive, it is possible to obtain InAs@ZnSe core@shell NCs in a one-pot approach, with photoluminescence (PL) quantum yield (PLQY) as high as ≈70% at 900 nm.^22,46^ However, the synthesis of large-sized amino-As-based InAs NCs emitting beyond 1000 nm in a one-pot approach, remains challenging. For instance, Leemans et al. showed that In­(As,P) NCs could exhibit an Abs peak up to ≈1600 nm when synthesized using amino phosphine as the reducing agent; however, PL results were not disclosed.^47^ Similarly, Kim et al. reported InAs NCs with an excitonic Abs peak up to 1700 nm but did not show any PL.^44^ In one of the few studies on this topic, Ginterseder et al. synthesized amino-As-based InAs NCs using an In­(I)Cl precursor acting as both the indium source and reducing agent. To obtain emission beyond 1000 nm, the NCs required the overgrowth of CdSe or CdS shells; however, these compositions are not RoHS-compliant.^48^ The only reported work that achieved efficient SWIR emission from amino-As-based InAs@ZnSe NCs was published by Panda et al.^38^ Yet, this method still relied on a complex continuous coinjection protocol.

These drawbacks have prompted us to devise an appropriate one-pot reaction route to synthesize cadmium-free and emissive large-sized amino-As-based InAs NCs , which we report in this work. This is achieved by employing a commercially available Grignard compound as a reducing agent in the synthesis of InAs NCs. Since their discovery in 1900, Grignard reagents have become one of the most widely used organometallic compounds, enabling the formation of carbon–carbon (C–C) bonds for the synthesis of organic molecules such as alcohols, ketones, and carboxylic acids.^49^ Grignard reagents feature the R-MX chemical formula, where X is a halide (Cl, Br, I), R is an alkyl, aryl, or vinyl group, and M is a metal, typically Mg or Zn. In this structure, the carbon atom connected to the metal atom has a partial negative charge, whereas the latter features a partial positive charge, making the carbon–metal bond (C^δ−^-M^δ+^) highly polar. Interestingly, by varying either the R- or the metal-halide groups, it is possible to modulate the reactivity of these reducing agents.^50^

Among the commercially available Grignard reagents, we selected benzyl magnesium chloride (BnMgCl) as a reducing agent as it is cost-effective and features high nucleophilicity; hence, it is effective at donating electrons pairs to an electrophile. Our results indicate that such reducing agent enables good control over the size of InAs NCs, from ≈3 nm to ≈4.2 nm, and optical Abs from 835 to 1180 nm. These NCs were subsequently coated with a ZnSe shell, leading to^22^ InAs@ZnSe core@shell NCs with PL tunable from 1000 to 1500 nm, and PLQYs as high as 46% at 1030 nm, 16% at 1190 nm, and 6% at 1500 nm. These efficiencies were achieved thanks to the compatibility of our one-pot synthesis route with the presence of ZnCl_2_ as an additive, which has been shown to boost the PLQY of the resulting amino-As-based InAs@ZnSe core@shell NCs.^46^

## Experimental Section

### Chemicals

Indium­(III) chloride (InCl_3_, 99.99%), zinc­(II) chloride (ZnCl_2_, 99.99%), benzyl magnesium chloride (BnMgCl, 2 M solution in tetrahydrofuran), 1-octadecene (ODE, 90%), and oleylamine (OLAM, 98%) were purchased from Sigma-Aldrich. Tri-n-octylphosphine (TOP, 97%), selenium powder (Se, 99.99%), and tris­(dimethylamino)­arsine (amino-As, 99%) were purchased from Strem Chemicals. Methyl magnesium iodide (3 M solution in diethyl ether), butyl magnesium chloride (2 M solution in THF), ethyl magnesium bromide (1 M solution in THF), 3-thienyl magnesium iodide (0.3 M solution in THF), 9-phenylnaphthyl magnesium bromide (0.5 M solution in THF), isopropyl magnesium chloride (2 M solution in THF), benzyl zinc bromide (0.5 M solution in THF), phenyl zinc bromide (0.5 M solution in THF), methyl magnesium bromide (3 M solution in diethyl ether), methyl zinc chloride (2 M solution in THF), vinyl magnesium chloride (1 M solution in THF), 3,4-difluorophenyl magnesium bromide (0.5 M solution in THF), toluene (anhydrous, 99.8%), tetrachloroethylene (TCE, 99%), and ethanol (anhydrous, 99.8%) were purchased from Sigma-Aldrich. All of the chemicals were used without further purification.

#### Preparation of ODE and OLAM Stock Solutions

In order to synthesize InAs core NCs, we first degassed 30 mL of ODE and OLAM at 120 °C for 2 h in a glovebox for further experiments.

#### Amino-As Precursor Preparation

For the QD-1 InAs core NCs, 0.2 mmol of amino-As was added into 0.5 mL of degassed OLAM and mixed for 5 min at 40 °C in a glovebox. Instead, to synthesize QD-2 and QD-3 cores, 0.4 mmol of amino-As was used, while all other steps were kept constant.

#### Preparation of ZnCl_2_-OLAM (0.8 M) Precursor

In the glovebox, 8 mmol of ZnCl_2_ was mixed with 10 mL of degassed OLAM at 250 °C for 1 h. Due to its solidification at room temperature, it needed to be preheated to around 120 °C before injection.

#### Preparation of TOP-Se (1 M) Precursor

In order to prepare a stock TOP-Se solution, 10 mmol of powdered selenium was mixed with 10 mL of trioctylphosphine (TOP) under constant stirring at 120 °C for 15 min. Then, it was cooled down to room temperature and kept in a glovebox for further experiments.

#### InAs Core NC Synthesis

InAs cores of three different sizes with optical Abs at 835, 990, and 1180 nm, and the corresponding InAs@ZnSe core@shell NCs, were synthesized through a modified literature method without any size-selective precipitation.^22^ First, to synthesize the QD-1 InAs core, a mixture of 0.2 mmol InCl_3_, 1 mmol ZnCl_2_, and 5 mL of degassed OLAM was kept under vacuum at 120 °C for 1 h. Then, 0.6 mmol (300 μL) BnMgCl was added to it at 120 °C under an inert atmosphere (Argon) and heated to 240 °C. Upon reaching 240 °C, 0.2 mmol amino-As (in 0.5 mL degassed OLAM) solution was quickly injected into the main solution and mixed at 300 °C for 15 min. Afterward, it was cooled down to room temperature. For the washing step, anhydrous toluene and ethanol were added and the resulting mixture was centrifuged at 6000 rpm for 3 min. For QD-2 and QD-3 InAs core NCs, a similar procedure was followed with minor modifications. In order to synthesize QD-2 InAs cores, a mixture of 0.4 mmol InCl_3_, 2 mmol ZnCl_2_, and 5 mL of a degassed OLAM + ODE mixture (OLAM:ODE ratio of 4:1) was kept under vacuum at 120 °C for 1 h. Afterward, 1.2 mmol (600 μL) BnMgCl was added to it at 120 °C under an inert atmosphere and heated to 240 °C. Similarly, an amino-As solution (0.4 mmol, in 0.5 mL degassed OLAM) was injected at 240 °C and heated to 300 °C. It was mixed for 5 min at that temperature. To precipitate the resulting NCs, the same aforementioned procedure was performed. For the synthesis of the QD-3 InAs cores, the same parameters were used as in the QD-2 cores synthesis, except that the OLAM:ODE ratio and the growth time of the cores were adjusted to 3:2 and 45 min, respectively. The final core NCs were dissolved in TCE for further optical characterization.

#### InAs@ZnSe Core@shell NC Synthesis

For the synthesis of QD-1 InAs@ZnSe core@shell NCs, instead of cooling down the crude solution of the QD-1 core to room temperature, it was cooled down to 90 °C. Subsequently, 2.5 mL ZnCl_2_–OLAM and 7.5 mL TOP-Se solutions were injected, and the temperature was raised to 300 °C. At that temperature, the crude solution was stirred for 3 h. The same procedure was performed for the synthesis of QD-2 and QD-3 core@shell samples, respectively. In this case, instead of stirring the crude solutions of QD-2 and QD-3 core@shell NCs for 3 h at 300 °C, they were stirred for 4 h at 300 °C. The resulting NCs were precipitated using anhydrous toluene and ethanol followed by centrifugation at 6000 rpm for 3 min. The purification step was repeated three times. The final core@shell NCs were dissolved in TCE for further optical characterization.

#### InAs Core NC Synthesis with Other Grignard Reagents

The synthesis of InAs core NCs using various other commercial Grignard reagents followed the same procedure as the one for the QD-1 core sample. Briefly, a mixture of 0.2 mmol InCl_3_, 1 mmol ZnCl_2_, and 5 mL of degassed OLAM was kept under vacuum at 120 °C for 1 h. Then, 0.6 mmol of the corresponding Grignard reagents was added to it and heated to 240 °C. At that temperature, 0.2 mmol amino-As (in 0.5 mL degassed OLAM) was quickly injected, after which the solution was heated up to 300 °C and stirred for 15 min. Afterward, the mixture was cooled down to room temperature and the NCs were precipitated by adding anhydrous toluene and ethanol followed by centrifugation at 6000 rpm for 3 min. The final NCs were dissolved in TCE for further measurements.

#### Optical Characterization

The optical Abs measurements of the resulting NCs were carried out on a Varian Cary 5000 UV–vis–NIR spectrophotometer. The steady-state and time-resolved PL measurements were carried out using an Edinburgh Instruments FLS900 fluorescence spectrometer equipped with an Xe lamp and a monochromator for steady-state PL excitation, and a time-correlated single-photon counting unit coupled with an Edinburgh Instruments EPL-510 pulsed laser diode (λ_ex_ = 508 nm, pulse width = 177 ps) for time-resolved PL. The PLQY measurements were performed using the Edinburgh Instruments FLS900 fluorescence spectrometer equipped with an integrating sphere and the output of an Xe lamp. The excitation wavelength for the QD-1 core@shell sample was 700 nm, whereas for the QD-2 and QD-3 core@shell samples, an 800 nm excitation wavelength was used. For the measurements, the samples were prepared by diluting the NC solutions in 3 mL of TCE in 1 cm path length quartz cuvettes in an N_2_-filled glovebox. All NC solutions were diluted to an optical density ≈0.1 at the excitation wavelength. Finally, it is important to underline that both water (i.e., air humidity) and OLAM (used as a surface ligand) have optical absorption features at wavelengths >1200 nm. Such features can affect the PL spectral shape, its intensity, and the PLQY.

#### Powder X-ray Diffraction (XRD) Analysis

XRD analysis was performed using a PANanalytical Empyrean X-ray diffractometer, equipped with a 1.8 kW Cu Kα ceramic X-ray tube and a PIXcel3D 2 × 2 area detector, operating at 45 kV and 40 mA. XRD samples were prepared by drop-casting highly concentrated solutions of NCs in anhydrous toluene onto a silicon wafer. All samples were prepared in a glovebox. XRD data were analyzed by the HighScore 4.1 software from PANalytical.

#### X-ray Photoelectron Spectroscopy (XPS) Analysis

XPS analyses were carried on a Kratos Axis Ultra^DLD^ spectrometer, using a monochromatic Al Kα source operated at 20 mA and 15 kV. XPS samples were prepared in an N_2_-filled glovebox by drop-casting a few microliters of highly concentrated NC solutions in anhydrous toluene onto a freshly cleaved, highly oriented pyrolytic graphite (HOPG) substrate. Survey scan analyses were carried out on an analysis area of 300 × 700 μm and a pass energy of 160 eV. High-resolution analyses were carried out with the same analysis area at a pass energy of 10 eV. The spectra were charge-corrected to the main line of the carbon 1s spectrum (adventitious carbon) set to 284.8 eV and were analyzed using the CasaXPS software (version 2.3.26).^51^

#### Transmission Electron Microscopy Characterization

Diluted InAs core and core@shell NCs solutions were drop-cast on copper transmission electron microscopy (TEM) grids coated with an ultrathin carbon film. Low-resolution TEM images were acquired using a JEOL JEM-1400Plus microscope with a thermionic gun (LaB_6_) operated at an acceleration voltage of 120 kV. High-resolution scanning transmission electron microscopy (STEM) images were acquired on a probe-corrected ThermoFisher Spectra 300 S/TEM operated at 300 kV. The images were acquired on a High-Angle Annular Dark Field (HAADF) detector with a current of 50 pA. Compositional maps were acquired using Velox, with a probe current of ≈150 pA and rapid rastered scanning of the beam. The Energy-Dispersive X-ray (EDX) signal was collected by a Dual-X system, comprising two detectors, one on either side of the sample, for a total acquisition solid angle of 1.76 sr.

#### Inductively Coupled Plasma (ICP) Analysis

The elemental analysis was also performed via inductively coupled plasma optical emission spectroscopy (ICP-OES) with an iCAP 6300 DUO ICP-OES spectrometer (ThermoScientific). The NC samples were digested in 1 mL of HNO_3_ overnight and then diluted with 9 mL of Milli-Q water for the measurements. The elemental analysis using ICP-OES was affected by a systematic error of ≈5%.

## Results and Discussion

The synthesis of InAs NCs with BnMgCl was carried out by employing the approach reported by Zhu et al. as a starting point.^45^ In detail, InAs NCs were synthesized from InCl_3_, ZnCl_2_, amino-As, OLAM, ODE, and BnMgCl. ZnCl_2_ and InCl_3_ (Zn:In ratio of 5:1) were dissolved in various amounts of OLAM and ODE, and the temperature was raised to 300 °C. The schematic representation of the procedure is illustrated in Figure 1a. Notably, metal–alkyl compounds in the presence of an alkylamine are known to react, forming the corresponding metal–alkylamide which, upon heating, can release hydride ions, as previously reported in the literature.^52–56^ Briefly, the metal–alkyl reagent, BnMgCl in the present case, reacts with OLAM, forming benzene and the corresponding metal-oleylamide (ClMg-oleylamide) (eq 1, M=MgCl, R_1_=C_17_H_33_ and R_2_=C_6_H_5_–CH_2_). Metal-oleylamides, upon heating, release hydride ions as a consequence of the oxidation of the oleylamide to *N*-oleyl–oleylimine (eq 2).^56^ Such hydride ions are believed to reduce As^3+^ ions and enable the formation of InAs NCs. We hypothesize that the rate at which the hydride ions are released by the Grignard reagents is slower than that of “standard” hydride-based reducing agents employed so far for the synthesis of InAs NCs, which include DMEA-AlH_3_, DIBAL-H, or superhydride, possibly explaining the different reaction outcome. Indeed, these compounds, can readily release H-species, and thus reduce In^3+^ and/or As^3+^ species, even at room temperature.^56^

1

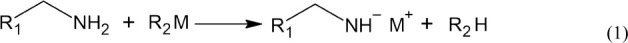



2

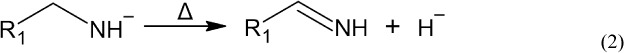




**Figure 1:**
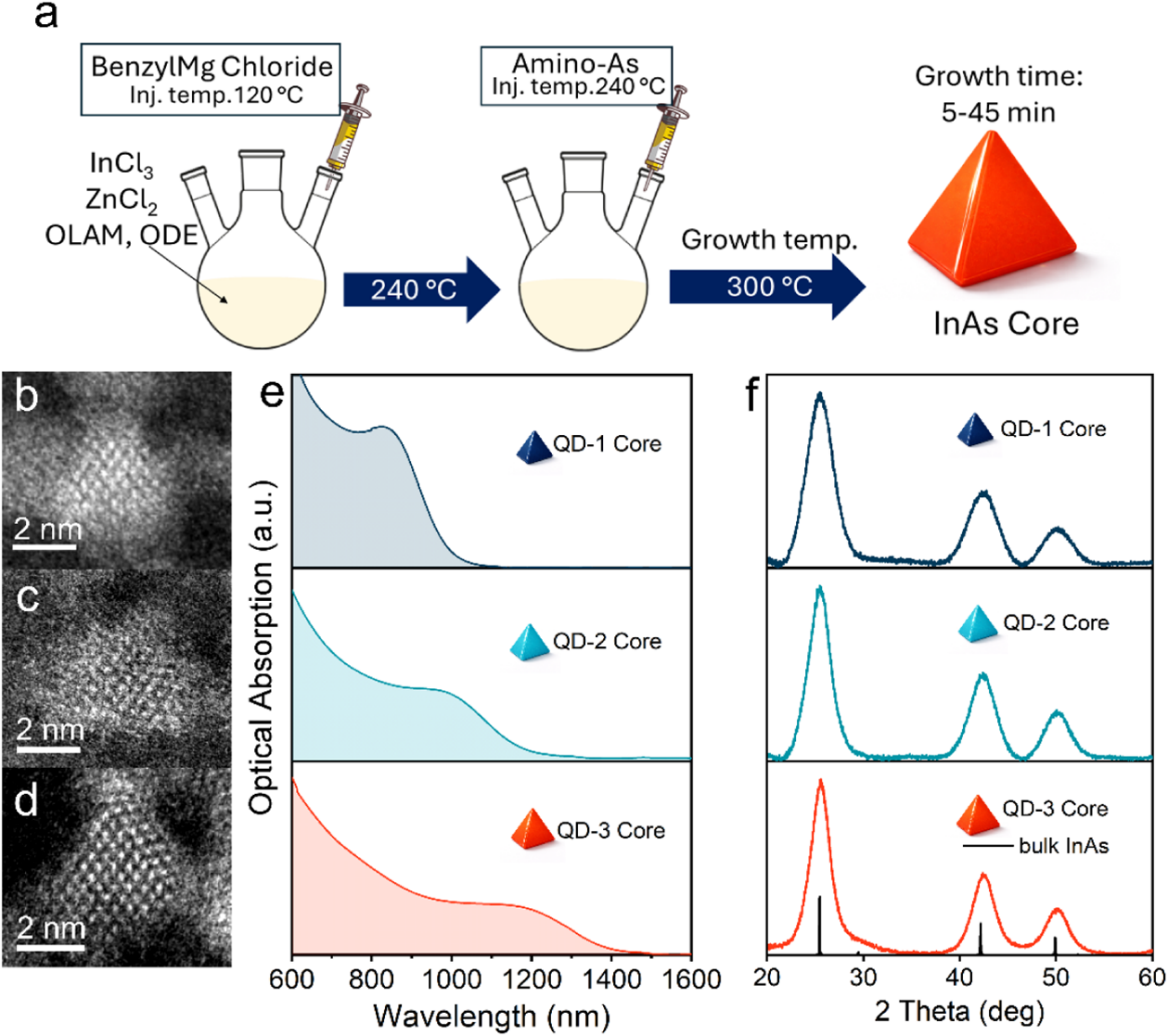
(a) Schematic representation of the synthesis of colloidal InAs core NCs. High-resolution high-angle annular dark field scanning electron microscopy (HR HAADF-STEM) images of the resulting InAs core NCs with sizes of (b) ≈3 nm (QD-1 core), (c) ≈3.4 nm (QD-2 core), and (d) ≈4.2 nm (QD-3 core), synthesized with different OLAM:ODE ratios and growth times. The scale bar is 2 nm. (e) Optical absorption spectra of colloidal InAs core NCs made with different OLAM:ODE ratios and reaction times, the optical absorption peaks are at 835, 990, and 1180 nm for QD-1core, QD-2 core, and QD-3 core, respectively. (f) XRD patterns of the InAs core NCs together with the bulk reflections of zinc-blende InAs (ICSD 98–002–4518). The absence of additional peaks shows that secondary phase formation did not occur in any of the samples.

Our results indicate that the use of OLAM as the sole solvent and a reaction time of 15 min led to the formation of InAs NCs (namely QD-1 core) with a size of ≈3 nm (Figure 1b). On the other hand, to synthesize large InAs core NCs, we used a OLAM/ODE mixture instead of just OLAM, and, in addition we had to vary the reaction time. The role of ODE is to increase the volume of the solvent in which the crystals nucleate and grow, and to lower the monomer concentration in solution, resulting in a larger critical radius of the starting nuclei.^47^ For details on the optimization studies related to the synthesis of NCs, see Experimental Section and Figures S1–S2, Tables S1–S2. In this regard, by using an OLAM:ODE volume ratio of 4:1 and a reaction time of 5 min, we could prepare InAs NCs with a size of ≈3.4 nm (QD-2 core), while a volume ratio of 3:2 combined with a reaction time of 45 min led to ≈4.2 nm (QD-3 core) NCs (Figure 1c-d and Figure S3). The QD-1 cores featured an optical Abs peak at 835 nm with a half-width at half-maximum (HWHM) of ≈140 meV, whereas the QD-2 and QD-3 core samples had an optical Abs peak centered at ≈990 nm with an HWHM of ≈190 meV and at ≈1180 nm with an HWHM of ≈200 meV, respectively (see Figure 1e). Notably, none of the three NC samples featured any PL emission, most likely due to the presence of unpassivated surface trap states, which are commonly found in amino-As-based InAs NCs.^38,41,48^

XRD analyses indicated that all three InAs NC samples had a zinc-blende crystal structure (ICSD 98–002–4518), with no presence of secondary phases. The increase in size from QD-1 to QD-3 core samples was corroborated by XRD, as narrower peak widths were observed going from sample QD-1 to QD-3 (Figure 1f). The elemental composition of the as-synthesized InAs core NCs, measured by XPS, revealed an In/As ratio slightly higher than 1 in all cases, suggesting that the NCs have an In-rich surface (Figure S4 and Table S3). The three NC samples displayed similar XPS features, with the In 3d_5/2_ peak centered at 444.3 ± 0.2 eV and the As 3d_5/2_ peak at 40.6 ± 0.2 eV, in line with previous reports.^45,57^ All samples exhibited low-intensity Zn signals (with a Zn 2p_3/2_ peak at approximately 1021.8 eV), which we attribute to Zn species located at the surface of the NCs, consistent with our previous works on related systems prepared with other synthetic routes.^45,46^ Interestingly, ICP-OES and XPS revealed the presence of Mg only in the QD-3 InAs cores, whereas no Mg was detected in QD-1 and QD-2 samples (Figure S4 and Table S3). Moreover, STEM-EDX mapping of sample QD-3 indicated that the Mg detected by the ensemble techniques originates from byproducts formed during the reaction rather than the NCs themselves (Figure S5).

We then proceeded with the growth of a ZnSe shell over the three InAs core NCs. This was done using a procedure previously reported by our group, which consists of adding solutions of ZnCl_2_/OLAM and TOP/Se to the crude InAs reaction solution at 90 °C, followed by heating the mixture to 300 °C and a reaction time up to 240 min (Figure 2a and see **Experimental Section** for further details).^22^ Upon ZnSe shell growth, we observed in all the three cases broadening of the optical Abs peak and the appearance of a PL peak, with QD-1 core@shell featuring an emission centered at ≈1030 nm, while for QD-2 and QD-3 core@shell NCs the PL emission was at ≈1190 nm and ≈1500 nm, respectively (Figure 2b). To optimize the PL efficiency of our core@shell NCs, we monitored in both cases the variation of the PLQY as a function of the ZnSe shell growth time. The QD-1 core@shell sample exhibited a maximum PLQY value of 46.0% after 180 min, while QD-2 and QD-3 core@shell samples reached maximum PLQY values of 16.7% and 6.3%, respectively, after 240 min (Figure 2c). Upon further shell growth, the PLQY decreased in all three cases. This drop in efficiency was ascribed to the formation of structural defects stemming from strain-induced relaxation^58,59^ (Figure 2c). TRPL measurements revealed double exponential decay dynamics for all InAs@ZnSe core@shell samples, with the average PL lifetime decreasing from ≈81 ns in the case of the QD-1 core@shell sample to ≈64 ns for QD-3 core@shell NCs (Figure 2d and Table S4).

**Figure 2:**
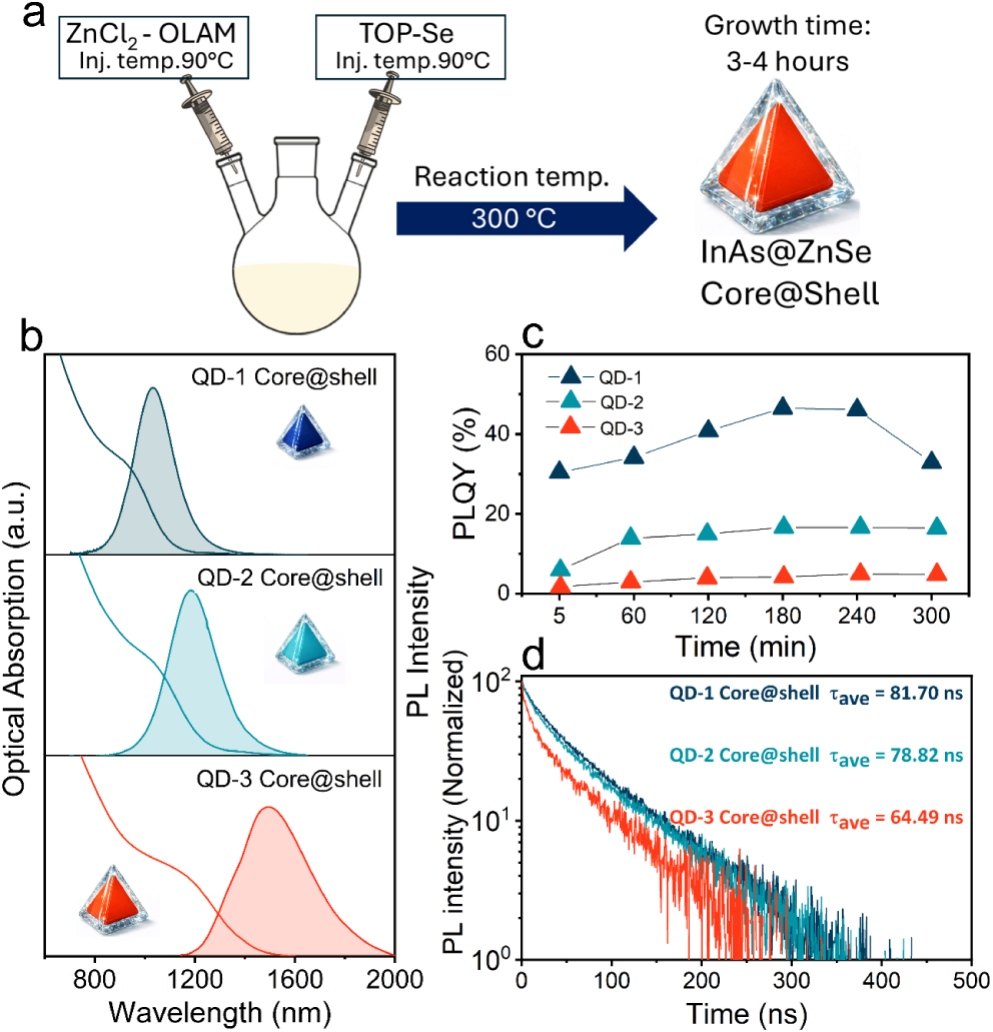
(a) Schematic representation of the synthesis of colloidal InAs@ZnSe NCs. (b) Optical absorption and PL spectra of InAs@ZnSe core@shell NCs emitting at ≈1030 nm (QD-1 core@shell), ≈1190 nm (QD-2 core@shell), and ≈1500 nm (QD-3 core@shell). (c) Variation of PLQY values of InAs@ZnSe core@shell NCs as a function of ZnSe shell growth time. (d) PL decay curves of InAs@ZnSe core@shell NCs.

The XRD patterns of the core@shell NCs corroborated the ZnSe shell formation, as indicated by the main diffraction peaks shifting toward higher two-theta angles, and excluded the presence of any undesired secondary phases (Figure S6). The XPS and ICP-OES analyses of the core@shell samples revealed In/As ratios higher than those of the starting InAs cores (Figure S7 and Table S5), suggesting the formation of an In–Zn–Se interlayer between the InAs core and the ZnSe shell, as already observed by us in previous studies.^22^ It is also worth highlighting that Mg was not observed in any of the core@shell samples, indicating that Mg is not able to form ZnMgSe under our reaction conditions. This is also in line with previously reported works (Table S5).^60^

Low-magnification TEM images, together with corresponding size distribution histograms, show that all core@shell NCs had a size of ≈10 nm after ZnSe shell growth, indicating a shell thickness of ≈6–7 monolayers (Figure 3a–c). High-resolution scanning electron microscopy high-angle annular dark field (HR STEM HAADF) images of core@shell NCs and EDX analyses are reported in Figure 3d–f and Figures S8–9, revealing that in all three cases, the InAs core was not positioned at the center of the core@shell structure; in fact, it was observed to be closer to one of the facets of the tetrahedron (Figure 3d–f), suggesting a preferential growth of ZnSe on specific facets of the InAs core. The off-center positioning of the core is affected by facet-specific surface energies, precursor reactivity, and thermodynamic-kinetic growth balance, as previously discussed by Li et al.^61^ In our case, in the presence of TOP, a thermodynamically controlled growth regime dominates, which minimizes the overall surface energy by enabling surface atoms to diffuse and reorganize during the ZnSe growth. As a result, it promotes inhomogeneous shell growth, which can lead to incomplete surface passivation.

**Figure 3:**
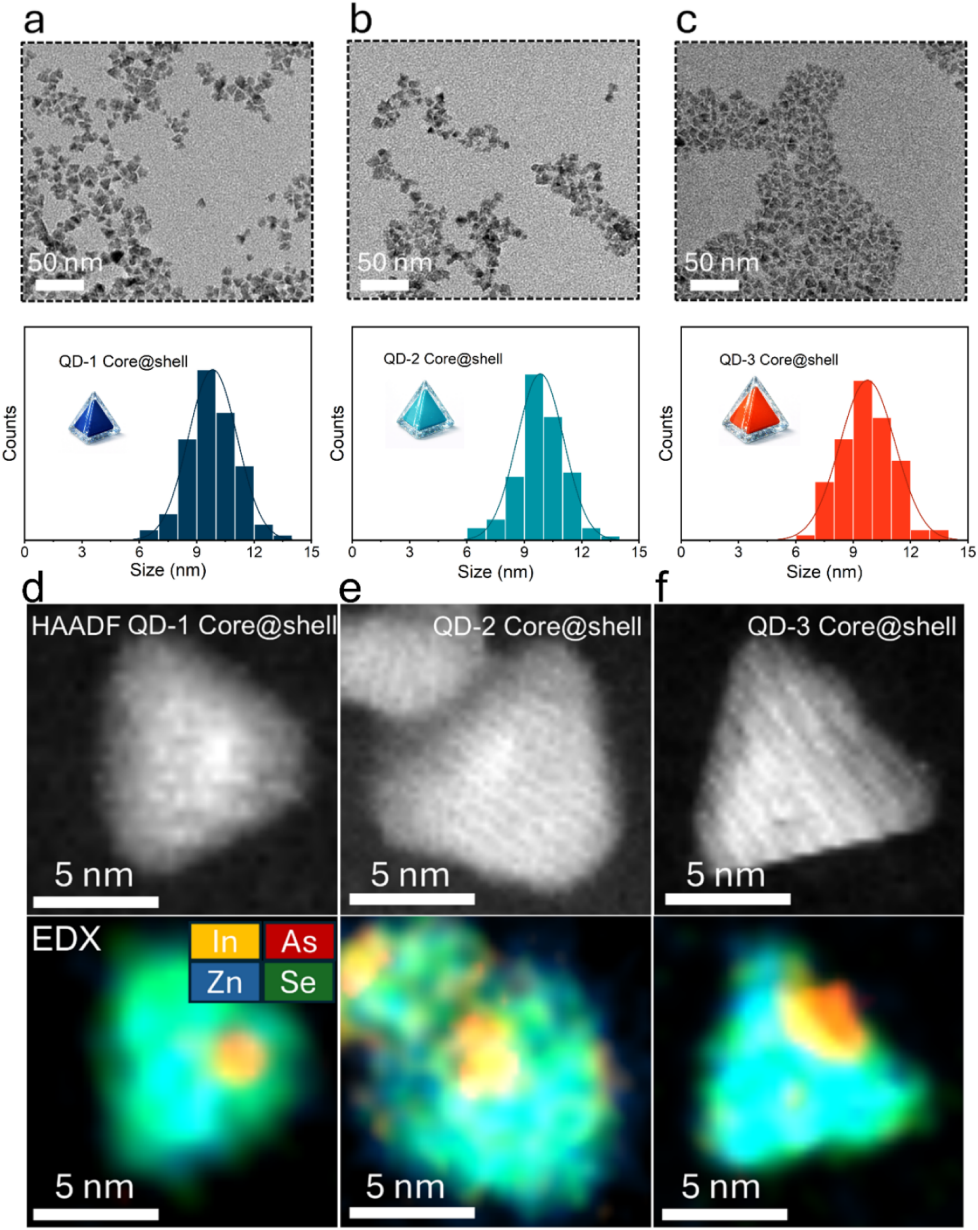
Low-resolution TEM images of InAs@ZnSe core@shell NCs with size distribution histograms: (a) QD-1 core@shell (9.9 ± 1.3 nm), (b) QD-2 core@shell (9.8 ± 1.2 nm), and (c) QD-3 core@shell NCs (9.7 ± 1.5 nm). HAADF-STEM images and corresponding EDX elemental maps of individual InAs@ZnSe core@shell NCs: (d) QD-1 core@shell, (e) QD-2 core@shell, and (f) QD-3 core@shell NCs.

The procedure we developed thus enables the one-pot synthesis of InAs NCs with PL tunability up to 1500 nm and with good PLQY values, paving the way for further developments. In this regard, we conducted a series of InAs NC syntheses to investigate the effect of the reactivity of the reducing reagent on InAs NC growth by employing 12 different commercially available Grignard reagents in addition to BnMgCl (Figure S10 and Table S6). All of these experiments with different Grignard reagents followed the same reaction parameters used to synthesize QD-1 InAs core NCs (Zn:In ratio, injection temperature, growth time, etc.). As shown in Figure S10, the resulting InAs core NCs synthesized using phenyl zinc bromide (PhZnBr) and vinyl magnesium bromide (ViMgBr) did not feature distinct optical Abs peaks (see Table S6). On the other hand, ethyl Mg chloride (experiment 3), 9-phenylnaphthyl Mg bromide (experiment 5), and 3,4-difluorophenyl Mg bromide (experiment 12) led to good control over the size and size distribution, similar to BnMgCl, thus also deserving potential future interest. Overall, these preliminary findings based on alternative Grignard reagents indicate that they can function as reducing agents for the synthesis of InAs-based NCs.

## Conclusions

In conclusion, we have reported an amino-As-based one-pot synthesis approach to size-tunable InAs NCs with emission reaching 1500 nm. The synthetic approach is based on a commercially available Grignard compound (benzylmagnesium chloride) as a reducing agent for InAs NCs, enabling the hot-injection synthesis of three differently sized InAs core NCs with optical Abs peaks ranging from 835 to 1180 nm, using amino-As and ZnCl_2_. Our structural characterization analysis confirmed the formation of InAs core NCs, with sizes ranging from ≈3 nm to ≈4.2 nm. To boost PL efficiency, we further grew a ZnSe shell onto all three InAs core NCs at high temperature. The resulting IR-emitting InAs@ZnSe core@shell NCs featured maximum PLQYs of ≈46%, ≈16%, and ≈6% at ≈1030, ≈1190, and ≈1500 nm emission, respectively. We further demonstrated that the synthesis of InAs NCs is compatible with a variety of commercially available Grignard reagents, encompassing different R-groups or metal-halides. Overall, our approach represents a significant starting point to synthesize IR-emitting InAs-based NCs with tunable PL emissions in one-pot by employing Grignard reagents as reducing agents.

## Supplementary Material



## Notes

Optical absorption spectra of InAs core NCs synthesized with different Zn:In and OLAM:ODE feed ratios; HAADF-STEM images and size distribution histograms of InAs core NCs; optical absorption spectra of as-synthesized InAs core NCs using various Grignard reagents; HAADF-STEM images and corresponding EDX elemental mapping of InAs@ZnSe core@shell NCs (QD-2 core@shell and QD-3 core@shell NCs); HAADF-STEM images and corresponding EDX elemental mapping of QD-3 InAs core NCs; HRSTEM images of individual InAs@ZnSe core@shell NCs (QD-1 core@shell and QD-3 core@shell); XPS spectra of InAs core (QD-1, QD-2, and QD-3) and InAs@ZnSe core@shell (QD-1 core@shell, QD-2 core@shell, QD-3 core@shell) NCs; ICP-OES analysis of InAs core (QD-1, QD-2, and QD-3) and InAs@ZnSe core@shell NCs (QD-1 core@shell, QD-2 core@shell, QD-3 core@shell); XRD patterns of InAs@ZnSe core@shell NCs; PL decay parameters of InAs@ZnSe core@shell NCs (PDF)

## ∇

Department of Materials Science and Engineering, Uppsala University, Uppsala, Sweden

## References

[1] Lu H., Carroll G. M., Neale N. R., Beard M. C. (2019). Infrared quantum
dots: Progress, challenges, and opportunities. ACS Nano.

[2] Jalali H. B., De Trizio L., Manna L., Di Stasio F. (2022). Indium arsenide
quantum dots: An alternative to lead-based infrared emitting nanomaterials. Chem. Soc. Rev..

[3] Müller T., Skiba-Szymanska J., Krysa A. B., Huwer J., Felle M., Anderson M., Stevenson R. M., Heffernan J., Ritchie D. A., Shields A. (2018). A quantum light-emitting diode for
the standard telecom window around 1,550 nm. Nat. Commun..

[4] De
Franco M., Zhu D., Asaithambi A., Prato M., Charalampous E., Christodoulou S., Kriegel I., De Trizio L., Manna L., Bahmani
Jalali H. (2022). Near-infrared light-emitting diodes based on RoHS-compliant
InAs/ZnSe colloidal quantum dots. ACS Energy
Lett..

[5] Roshan H., Mazza D., Panda S., de Boni F., De Trizio L., Manna L., Di Stasio F. (2026). Short-Wave Infrared InAs Quantum-Dot
Light-Emitting Diodes with Tunable Electroluminescence beyond 1.4
μm. ACS Energy Lett..

[6] Roshan H., Zhu D., Piccinotti D., Dai J., De Franco M., Barelli M., Prato M., De Trizio L., Manna L., Di Stasio F. (2024). Near Infrared Light-Emitting Diodes
Based on Colloidal InAs/ZnSe Core/Thick-Shell Quantum Dots. Adv. Sci.

[7] Chen J., Zheng S., Jia D., Liu W., Andruszkiewicz A., Qin C., Yu M., Liu J., Johansson E. M., Zhang X. (2021). Regulating thiol ligands of p-type
colloidal quantum dots for efficient
infrared solar cells. ACS Energy Lett.

[8] Pejović V., Georgitzikis E., Lee J., Lieberman I., Cheyns D., Heremans P., Malinowski P. E. (2022). Infrared
colloidal quantum dot image sensors. IEEE Trans.
Electron. Devices.

[9] Zhou R., Xu J., Luo P., Hu L., Pan X., Xu J., Jiang Y., Wang L. (2021). Near-infrared
photoactive semiconductor
quantum dots for solar cells. Adv. Energy Mater.

[10] Ahn N., Livache C., Pinchetti V., Jung H., Jin H., Hahm D., Park Y.-S., Klimov V. I. (2023). Electrically driven
amplified spontaneous emission from colloidal quantum dots. Nature.

[11] Christodoulou S., Ramiro I., Othonos A., Figueroba A., Dalmases M., Özdemir O., Pradhan S., Itskos G., Konstantatos G. (2020). Single-exciton
gain and stimulated emission across
the infrared telecom band from robust heavily doped PbS colloidal
quantum dots. Nano Lett.

[12] Kwon H.-J., Lee S.-H. (2021). Visible and near-infrared
image acquisition and fusion
for night surveillance. Chemosensors.

[13] Medintz I. L., Uyeda H. T., Goldman E. R., Mattoussi H. (2005). Quantum dot
bioconjugates for imaging, labelling and sensing. Nat. Mater.

[14] Zhang Z., Chang H., Xue B., Zhang S., Li X., Wong W.-K., Li K., Zhu X. (2018). Near-infrared and visible
dual emissive transparent nanopaper based on Yb (III)–carbon
quantum dots grafted oxidized nanofibrillated cellulose for anti-counterfeiting
applications. Cellulose.

[15] Gréboval C., Chu A., Goubet N., Livache C., Ithurria S., Lhuillier E. (2021). Mercury chalcogenide
quantum dots: Material perspective for device integration. Chem. Rev.

[16] Keuleyan S., Lhuillier E., Guyot-Sionnest P. (2011). Synthesis of colloidal HgTe quantum
dots for narrow mid-IR emission and detection. J. Am. Chem. Soc.

[17] Deng Y. H., Pang C., Kheradmand E., Leemans J., Bai J., Minjauw M., Liu J., Molkens K., Beeckman J., Detavernier C. (2024). Short-wave infrared colloidal QD photodetector
with nanosecond response times enabled by ultrathin absorber layers. Adv. Mater.

[18] European Union Directive 2011/83/EU of the European Parliament and of the Council of 25 October 2011 on consumer rights, amending Council Directive 93/13/EEC and Directive 1999/44/EC of the European Parliament and of the Council and repealing Council Directive 85/577/EEC and Directive 97/7/EC of the European Parliament and of the Council Text with EEA relevance European Union 2022

[19] Yang L., Zhang S., Xu B., Jiang J., Cai B., Lv X., Zou Y., Fan Z., Yang H., Zeng H. (2023). I–III–VI
quantum dots and derivatives: Design, synthesis, and properties for
light-emitting diodes. Nano Lett.

[20] Wang Y., Peng L., Schreier J., Bi Y., Black A., Malla A., Goossens S., Konstantatos G. (2024). Silver telluride
colloidal quantum dot infrared photodetectors and image sensors. Nat. Photonics.

[21] Kwon Y., Yeromina O., Cavallo M., Silly M. G., Pierucci D., Lhuillier E., Aldakov D., Hyot B., Reiss P. (2024). Synthesis
of NIR/SWIR absorbing InSb nanocrystals using Indium (I) halide and
aminostibine precursors. Adv. Funct. Mater.

[22] Zhu D., Bahmani Jalali H., Saleh G., Di Stasio F., Prato M., Polykarpou N., Othonos A., Christodoulou S., Ivanov Y. P., Divitini G. (2023). Boosting the Photoluminescence
Efficiency of InAs Nanocrystals Synthesized with Aminoarsine via a
ZnSe Thick-Shell Overgrowth. Adv. Mater.

[23] Peng L., Wang Y., Ren Y., Wang Z., Cao P., Konstantatos G. (2024). InSb/InP Core–Shell Colloidal Quantum Dots for
Sensitive and Fast Short-Wave Infrared Photodetectors. ACS Nano.

[24] Kim T.-G., Zherebetskyy D., Bekenstein Y., Oh M. H., Wang L.-W., Jang E., Alivisatos A. P. (2018). Trap passivation in indium-based
quantum dots through surface fluorination: Mechanism and applications. ACS Nano.

[25] Reiss P., Carriere M., Lincheneau C., Vaure L., Tamang S. (2016). Synthesis
of semiconductor nanocrystals, focusing on nontoxic and earth-abundant
materials. Chem. Rev.

[26] Franke D., Harris D. K., Chen O., Bruns O. T., Carr J. A., Wilson M. W., Bawendi M. G. (2016). Continuous
injection synthesis of
indium arsenide quantum dots emissive in the short-wavelength infrared. Nat. Commun.

[27] Tamang S., Lee S., Choi H., Jeong S. (2016). Tuning size and size distribution
of colloidal InAs nanocrystals via continuous supply of prenucleation
clusters on nanocrystal seeds. Chem. Mater.

[28] Srivastava V., Janke E. M., Diroll B. T., Schaller R. D., Talapin D. V. (2016). Facile,
economic and size-tunable synthesis of metal arsenide nanocrystals. Chem. Mater.

[29] Kim T., Park S., Jeong S. (2021). Diffusion
dynamics controlled colloidal
synthesis of highly monodisperse InAs nanocrystals. Nat. Commun.

[30] Skorotetcky M. S., Mir W. J., Sheikh T., Yorov K. E., Saidzhonov B. M., Daws S., Zhou R., Hedhili M. N., Abulikemu M., Mohammed O. F. (2025). Si-H
Hydrosilane Reducing Agents for Size-
and Shape-Controlled InAs Colloidal Quantum Dots. Adv. Mater.

[31] Ban H. W., Vafaie M., Levina L., Xia P., Imran M., Liu Y., Najarian A. M., Sargent E. H. (2024). Resurfacing
of InAs colloidal quantum
Dots equalizes photodetector performance across synthetic routes. J. Am. Chem. Soc.

[32] Xia P., Wang S., Chen Y., Gulsaran A., Zhang Y., Vafaie M., Imran M., Najarian A. M., Liu Y., Ban H. (2025). Improved
Facet and Edge Passivation in Near-Infrared
III-V Colloidal Quantum Dot Photodetectors. Adv. Mater.

[33] Franke D., Harris D. K., Xie L., Jensen K. F., Bawendi M. G. (2015). The unexpected
influence of precursor conversion rate in the synthesis of III–V
quantum dots. Angew. Chem.

[34] Panda S., Sinatra L., Yorov K. E., Suwito G. R., Bessonov A., Lutfullin M., Goldoni L., Bergamaschi E., Brescia R., Prato M., Divitini G. (2025). Trioctylamine
in the Synthesis of Tris (trimethylsilyl) arsine-Based InAs Quantum
Dots Prevents the Formation of Si-Based Byproducts. J. Am. Chem. Soc..

[35] Zhao T., Oh N., Jishkariani D., Zhang M., Wang H., Li N., Lee J. D., Zeng C., Muduli M., Choi H.-J. (2019). General Synthetic Route to High-Quality Colloidal III–V Semiconductor
Quantum Dots Based on Pnictogen Chlorides. J.
Am. Chem. Soc..

[36] Sheikh T., Mir W. J., Nematulloev S., Maity P., Yorov K. E., Hedhili M. N., Emwas A.-H., Khan M. S., Abulikemu M., Mohammed O. F. (2023). InAs Nanorod Colloidal Quantum Dots with Tunable
Bandgaps Deep into the Short-Wave Infrared. ACS Nano.

[37] Uesugi H., Kita M., Omata T. (2014). Facile synthesis of colloidal InAs
nanocrystals using triphenylarsine as an arsenic source. J. Cryst. Growth.

[38] Panda S., Zhu D., Goldoni L., Asaithambi A., Brescia R., Saleh G., De Trizio L., Manna L. (2025). Overcoming the Short-Wave Infrared
Barrier in the Photoluminescence of Amino-As-Based InAs Quantum Dots. Adv. Opt. Mater..

[39] Antanovich A., Shamraienko V., Lauth J., Lesnyak V. (2025). Tribenzoylarsine –
A Benign Arsenic Precursor for InAs Nanocrystals. Small.

[40] Nedelcu G., Yakunin S., Yanchak A., Driess M., Grützmacher H., Dirin D. N., Kovalenko M. V. (2025). Air-Stable
Aluminum Tris [Bis­(mesitoyl)­Arsenide]
Precursor for Metal Arsenide Quantum Dots. Helv.
Chim. Acta.

[41] Grigel V., Dupont D., De Nolf K., Hens Z., Tessier M. D. (2016). InAs colloidal
quantum dots synthesis via aminopnictogen precursor chemistry. J. Am. Chem. Soc..

[42] Lee J., Zhao T., Yang S., Muduli M., Murray C. B., Kagan C. R. (2024). One-pot heat-up
synthesis of short-wavelength infrared,
colloidal InAs quantum dots. J. Chem. Phys..

[43] Srivastava V., Dunietz E., Kamysbayev V., Anderson J. S., Talapin D. V. (2018). Monodisperse
InAs quantum dots from aminoarsine precursors: Understanding the role
of reducing agent. Chem. Mater..

[44] Kim M., Lee J., Jung J., Shin D., Kim J., Cho E., Xing Y., Jeong H., Park S., Oh S. H. (2024). Surface-Originated Weak Confinement in Tetrahedral Indium Arsenide
Quantum Dots. J. Am. Chem. Soc..

[45] Zhu D., Bellato F., Bahmani
Jalali H., Di Stasio F., Prato M., Ivanov Y. P., Divitini G., Infante I., De Trizio L., Manna L. (2022). ZnCl_2_ Mediated synthesis
of InAs nanocrystals with aminoarsine. J. Am.
Chem. Soc..

[46] Zhu D., Llusar J., Asaithambi A., Liu Z., Bes R., Prieur D., Karakkal H. H., Prato M., Brovelli S., Saleh G. (2025). Unveiling the Role of ZnCl2 in Enhancing the Photoluminescence
Efficiency of Amino-As-Based InAs@ZnSe Quantum Dots. ACS Nano.

[47] Leemans J., Respekta D., Bai J., Braeuer S., Vanhaecke F., Hens Z. (2023). Formation of colloidal In (As, P) quantum Dots active in the Short-Wave
infrared, promoting growth through temperature ramps. ACS Nano.

[48] Ginterseder M., Franke D., Perkinson C. F., Wang L., Hansen E. C., Bawendi M. G. (2020). Scalable synthesis
of InAs quantum dots mediated through
indium redox chemistry. J. Am. Chem. Soc..

[49] Seyferth D. (2009). The grignard
reagents. ACS Publications.

[50] Smith, M. B. ; March, J. March’s advanced organic chemistry: Reactions, mechanisms, and structure; John Wiley & Sons, 2025.

[51] Fairley N., Fernandez V., Richard-Plouet M., Guillot-Deudon C., Walton J., Smith E., Flahaut D., Greiner M., Biesinger M., Tougaard S. (2021). Systematic
and collaborative
approach to problem solving using X-ray photoelectron spectroscopy. Appl. Surf. Sci. Adv..

[52] Miller R. C., Neilson J. R., Prieto A. L. (2020). Amide-assisted
synthesis of iron
germanium sulfide (Fe2GeS4) nanostars: The effect of LiN (SiMe3) 2
on precursor reactivity for favoring nanoparticle nucleation or growth. J. Am. Chem. Soc..

[53] He M., Protesescu L., Caputo R., Krumeich F., Kovalenko M. V. (2015). A general
synthesis strategy for monodisperse metallic and metalloid nanoparticles
(In, Ga, Bi, Sb, Zn, Cu, Sn, and their alloys) via in situ formed
metal long-chain amides. Chem. Mater..

[54] Kale A. R., Bullett W. E., Prieto A. L. (2023). Controlling
Phase Conversion of Cu-Sb-Se
Nanoparticles through the Use of an Amide Base. Nano Lett..

[55] Chen Y., Landes N. T., Little D. J., Beaulac R. (2018). Conversion
Mechanism
of Soluble Alkylamide Precursors for the Synthesis of Colloidal Nitride
Nanomaterials. J. Am. Chem. Soc..

[56] Kim H., Kim M., Shim D., Sabisch S., Jang E., Choi M., Park S., Xing Y., Oh S. H., Kovalenko M. V. (2026). Metal–Amide Chemistry Enables Controlled Heavy-Pnictogen Reduction
for Colloidal III–V Nanocrystal Synthesis. J. Am. Chem. Soc..

[57] Liu Z., Llusar J., Karakkal H. H., Zhu D., Ivanov Y. P., Prato M., Divitini G., Brovelli S., Infante I., De Trizio L. (2024). Amino-Arsine and Amino-Phosphine
Based Synthesis
of InAs@ InP@ ZnSe core@ shell@ shell Quantum Dots. Adv. Energy Mater..

[58] Cao, Banin U. (2000). Growth and properties
of semiconductor core/shell nanocrystals with InAs cores. J. Am. Chem. Soc..

[59] Peng X., Schlamp M. C., Kadavanich A. V., Alivisatos A. P. (1997). Epitaxial
growth of highly luminescent CdSe/CdS core/shell nanocrystals with
photostability and electronic accessibility. J. Am. Chem. Soc..

[60] Mulder J. T., Kirkwood N., De Trizio L., Li C., Bals S., Manna L., Houtepen A. J. (2020). Developing lattice
matched ZnMgSe
shells on InZnP quantum dots for phosphor applications. ACS Appl. Nano Mater..

[61] Li X., Scharf E., Levi A., Deree Y., Stone D., Remennik S., Banin U. (2025). Shell Phase
and Morphology Control
for Emission Tuning in III–V Core/Shell Quantum Dots. ACS Nano.

