# Supplementary material for: Large Colloidal InAs Nanocrystals Synthesized with a Grignard Reagent
Source: Chem Mater. 2026 Jun 12;38(13):6508–16. doi: 10.1021/acs.chemmater.6c00517 (PMC13374076; doi:10.1021/acs.chemmater.6c00517)
Supplement: Supplementary file 1 [file cm6c00517_si_001.pdf]

Supporting Information

# Large Colloidal InAs Nanocrystals Synthesized with a Grignard Reagent

Guncem Ozgun Eren[1], Houman Bahmani Jalali[1,#], Hossein Roshan[2], Mauro Garbarino[1,3], Yurii P. Ivanov[4], Mirko Prato[5], Giorgio Divitini[4], Luca De Trizio[6], Liberato Manna[2,*], Francesco Di Stasio[1,*]

1. Photonic Nanomaterials, Istituto Italiano di Tecnologia, 16163 Genova, Italy
2. Nanochemistry, Istituto Italiano di Tecnologia, 16163 Genova, Italy
3. Dipartimento di Chimica e Chimica Industriale, Università degli Studi di Genova, 16146 Genova, Italy
4. Electron Spectroscopy and Nanoscopy, Istituto Italiano di Tecnologia, 16163 Genova, Italy
5. Materials Characterization, Istituto Italiano di Tecnologia, 16163 Genova, Italy
6. Chemistry Facility, Istituto Italiano di Tecnologia, 16163 Genova, Italy

# Current Address: Department of Materials Science and Engineering, Uppsala University, Uppsala, Sweden

Corresponding Author Email: liberato.manna@iit.it, francesco.distasio@iit.it

**Optimization of Zn:In feed and OLAM:ODE volume ratios for InAs core NCs**

The InAs core synthesis was carried out using different Zn:In feed ratio ranging from 5:1 to 25:1. All the other reaction parameters were not varied. In a typical synthesis, a mixture of 0.2 mmol $InCl_3$, certain amount of $ZnCl_2$ based on Zn:In ratio, and 5 ml of degassed oleylamine (OLAM) were kept under vacuum at 120 °C for 1 h. Then, 300 µl Benzyl Magnesium Chloride (BnMgCl) was added to the mixture and heated to 240 °C. Upon reaching 240 °C, 0.2 mmol amino-As (in 0.5 ml degassed OLAM) solution was quickly injected into it and stirred at 300 °C for 15 min. For the centrifugation step, anhydrous toluene and ethanol were used. The centrifugation procedure was performed at 6000 rpm for 3 min. The optical absorption (Abs) spectra of the resulting InAs NCs is given in **Figure S1**. In order to obtain large InAs core NCs, we also investigated the optimum combination of OLAM and 1-octadecene (ODE) by varying their volume ratio ranging from 5:0 to 1:4 (OLAM:ODE). 1-octadecene, together with precursor concentrations, plays an important role to obtain large NCs as previously reported in literature[1, 2]. To this end, a mixture of $InCl_3$:amino-As: BnMgCl with a molar ratio of 1:1:3 was used in OLAM-ODE with varying OLAM:ODE volume ratio. Similar to previous centrifugation procedures, the resulting products were purified using anhydrous toluene and ethanol then centrifuged at 6000 rpm for 3 min. Suboptimal size control over the InAs NCs has been observed with increasing ODE ratio (**Figure S2**).

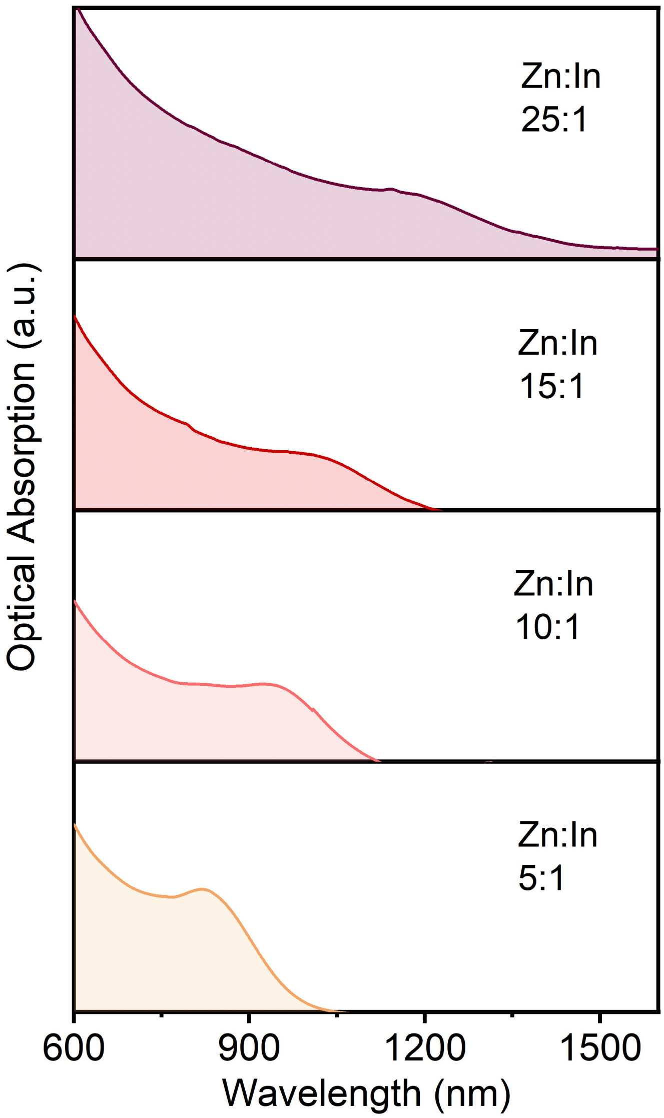


**Figure S1.** Optical Absorption spectra of InAs core NCs synthesized by different Zn:In feed ratio. All the other reaction parameters were kept constant: 0.2 mmol $InCl_3$ and amino-As, 5 ml OLAM, and 0.6 mmol BnMgCl. As the Zn:In ratio increased from 5:1 to 25:1, a red-shift of the Abs spectrum from 825 nm to 1190 nm occurred. The distinct excitonic peak at 825 nm with a HWHM of 120 meV also became less pronounced.

**Table S1.** Optical Abs and HMWM values of InAs core NCs synthesized with varying Zn:In feed ratio.

| Zn:In feed ratio (mmol) | Abs (nm) | HWHM (meV) |
|---|---|---|
| 5:1 | 825 | 136 |
| 10:1 | 940 | 215 |
| 15:1 | 1020 | 224 |
| 25:1 | 1190 | >250 |

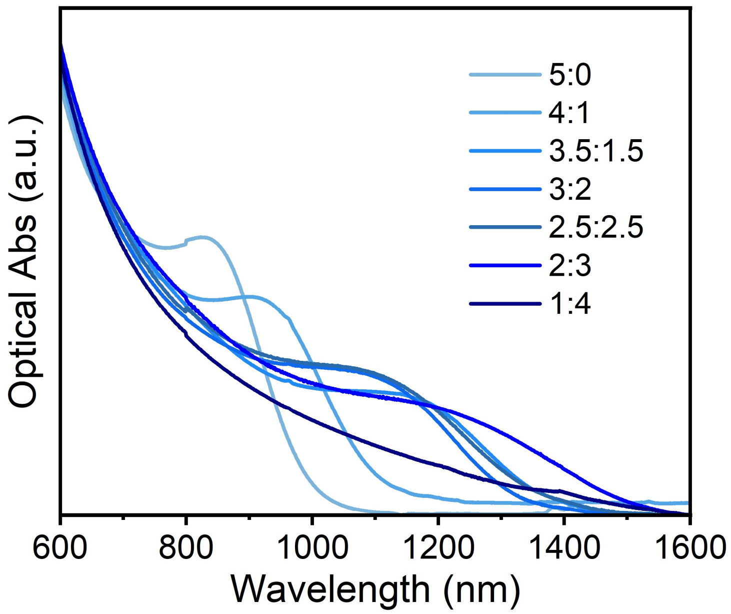


**Figure S2.** Optical absorption spectra of InAs core NCs synthesized with various OLAM:ODE ratios. While the combined use of OLAM and ODE resulted in obtaining large core NCs compared to OLAM solvent alone, poor control over the size was observed as the ODE ratio increased.

**Table S2**. Optical Abs and HMWM values of InAs core NCs which were synthesized by different OLAM:ODE volume ratio.

| OLAM:ODE volume ratio | Abs (nm) | HWHM (meV) |
|---|---|---|
| 5:0 | 830 | 122 |
| 4:1 | 910 | 180 |
| 3.5:1.5 | 1160 | 205 |
| 3:2 | 1110 | 223 |
| 2.5:2.5 | 1115 | 247 |
| 2:3 | 1235 | >250 |
| 1:4 | ----- | ----- |

**Characterization of InAs core NCs**

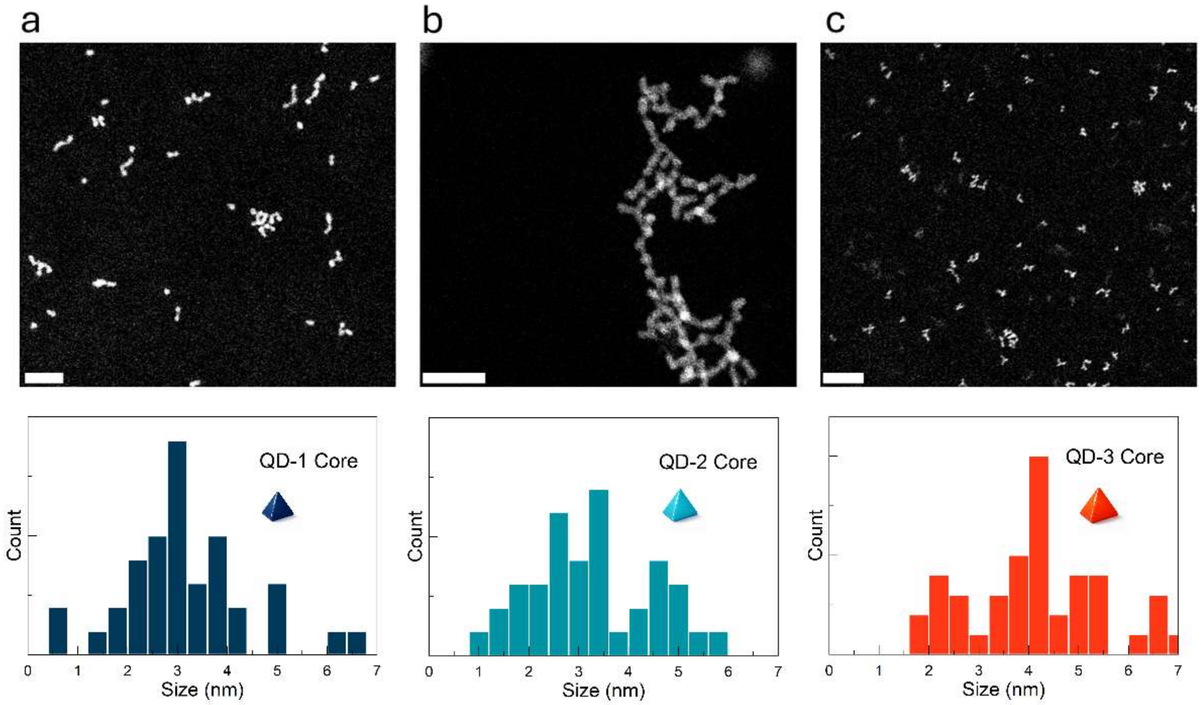


**Figure S3.** HAADF-STEM images and size distribution histograms of the as-synthesized (a) QD-1, (b) QD-2, and (c) QD-3 InAs core NCs. Scale bars are 20 nm for (a)-(b) and 50 nm for (c), respectively.

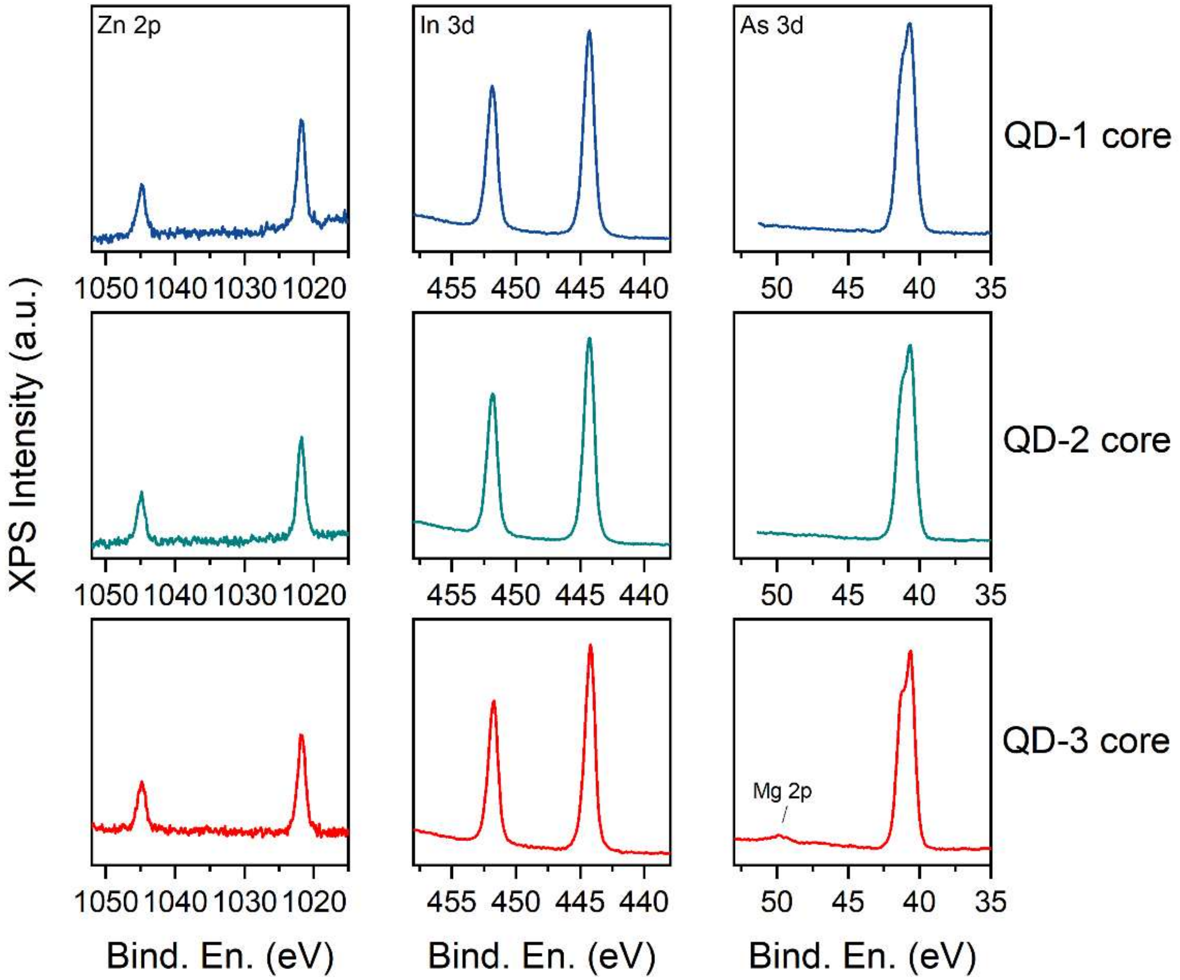


**Figure S4.** XPS analysis of QD-1, QD-2, and QD-3 InAs core NCs.

**Table S3.** XPS and ICP-OES results of InAs core NCs.

| **Sample** | ICP-OES | | | | XPS | | | |
|---|---|---|---|---|---|---|---|---|
| | In/As | Zn/As | Zn/In | Mg/In | In/As | Zn/As | Zn/In | Mg/In |
| **QD-1** | 1.18 | 0.06 | 0.05 | 0.01 | 1.12 | 0.04 | 0.03 | 0.00 |
| **QD-2** | 1.19 | 0.04 | 0.04 | 0.02 | 1.15 | 0.04 | 0.03 | 0.00 |
| **QD-3** | 1.25 | 0.003 | 0.004 | 0.21 | 1.11 | 0.04 | 0.04 | 0.12 |

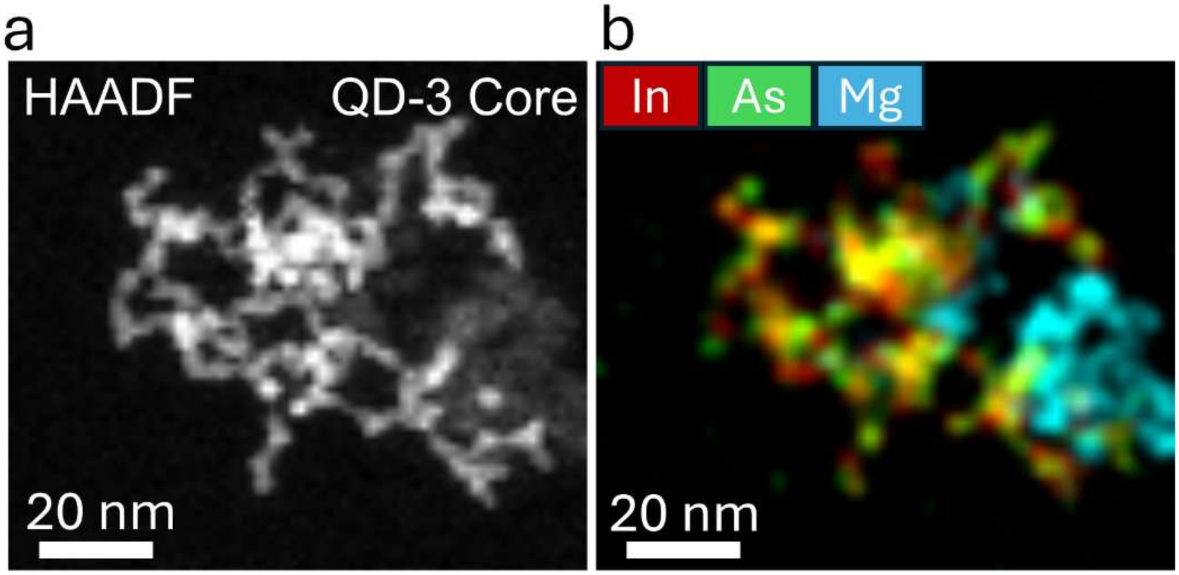


**Figure S5.** (a) and (b). STEM-EDX mapping of QD-3 InAs core NCs.

## Characterization of InAs@ZnSe core@shell NCs

**Table S4.** PL decay parameters of InAs@ZnSe core@shell NCs.

| Sample | A | $B_1$ | $\tau_1$(ns) | $B_2$ | $\tau_2$(ns) | $\tau_{ave}$(ns) |
|---|---|---|---|---|---|---|
| **QD-1 Core@shell** | 0.05 | 0.34 | 17.52 | 0.61 | 88.66 | 81.70 |
| **QD-2 Core@shell** | 0.04 | 0.39 | 14.87 | 0.57 | 86.26 | 78.82 |
| **QD-3 Core@shell** | 0.06 | 0.46 | 7.89 | 0.48 | 70.58 | 64.49 |

The decays were fitted with a bi-exponential decay function:

$Y = A+B_1*\exp(-t/\tau_1) +B_2*\exp(-t/\tau_2)$ $B_i$ and $\tau_i$ are amplitudes and PL lifetime, respectively.

The average lifetime ($\tau_{ave}$) was calculated by the function:

$\tau_{ave} = (B_1* \tau_1^2 + B_2* \tau_2^2)/( B_1* \tau_1+ B_2* \tau_2)$

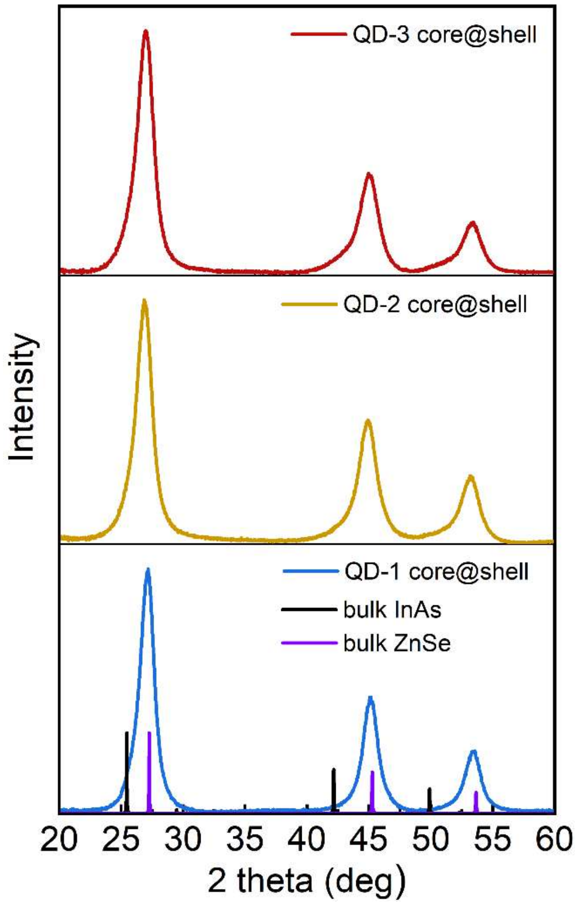


**Figure S6.** XRD patterns of core@shell NCs.

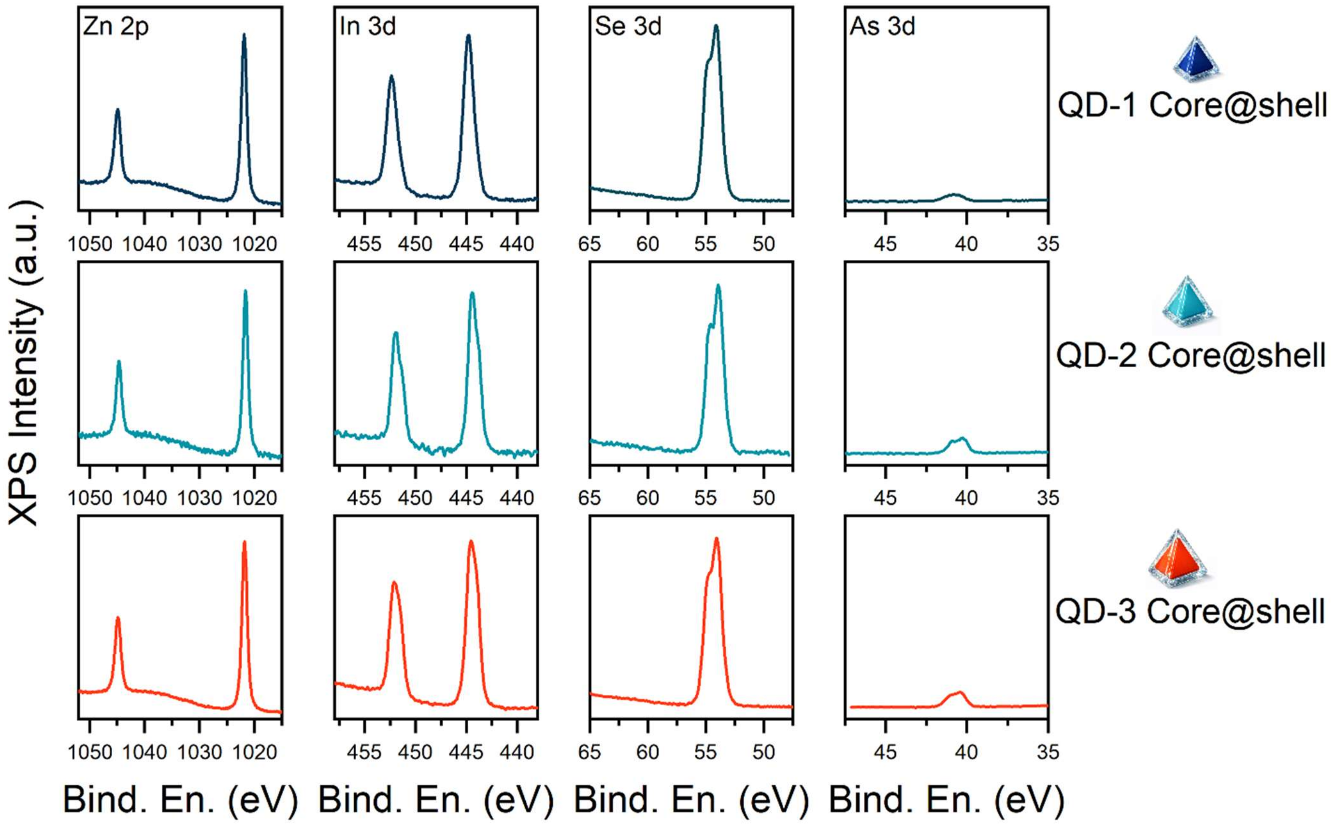


**FigureS7.** XPS analysis of InAs@ZnSe core@shell NCs.

**Table S5.** XPS and ICP-OES results of InAs@ZnSe core@shell NCs.

| **Sample** | **ICP-OES** | | | | | **XPS** | | | | |
|---|---|---|---|---|---|---|---|---|---|---|
| | In/As | Zn/As | Zn/In | Zn/Se | Mg/In | In/As | Zn/As | Zn/In | Zn/Se | Mg/In |
| **QD-1 Core@shell** | 1.5 | 25 | 16.66 | 1 | 0.00 | 4.46 | 26.05 | 5.85 | 1.06 | 0.00 |
| **QD-2 Core@shell** | 2.0 | 9.5 | 4.75 | 0.86 | 0.00 | 2.53 | 10.92 | 4.31 | 1.03 | 0.00 |
| **QD-3 Core@shell** | 2 | 10 | 5 | 0.90 | 0.00 | 2.40 | 11.92 | 4.98 | 1.09 | 0.00 |

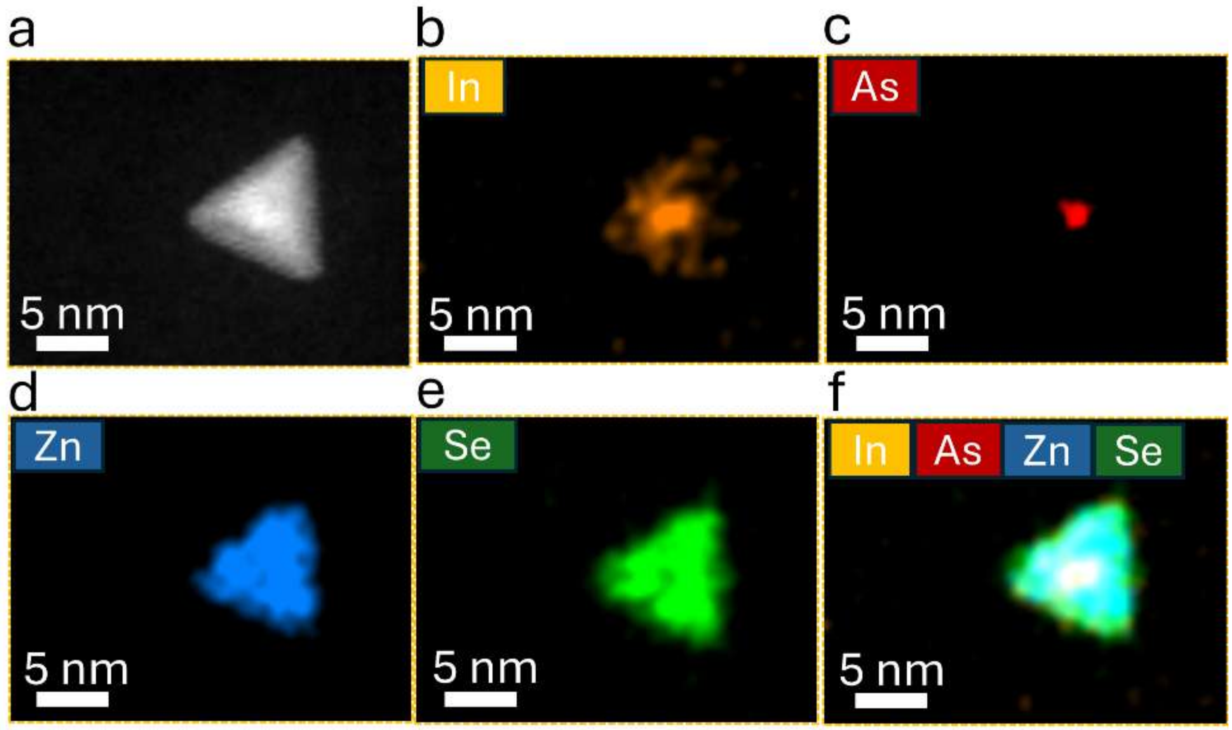


**Figure S8.** EDX elemental mapping of the resulting QD-2 core@shell NCs. The scale bar is 5 nm.

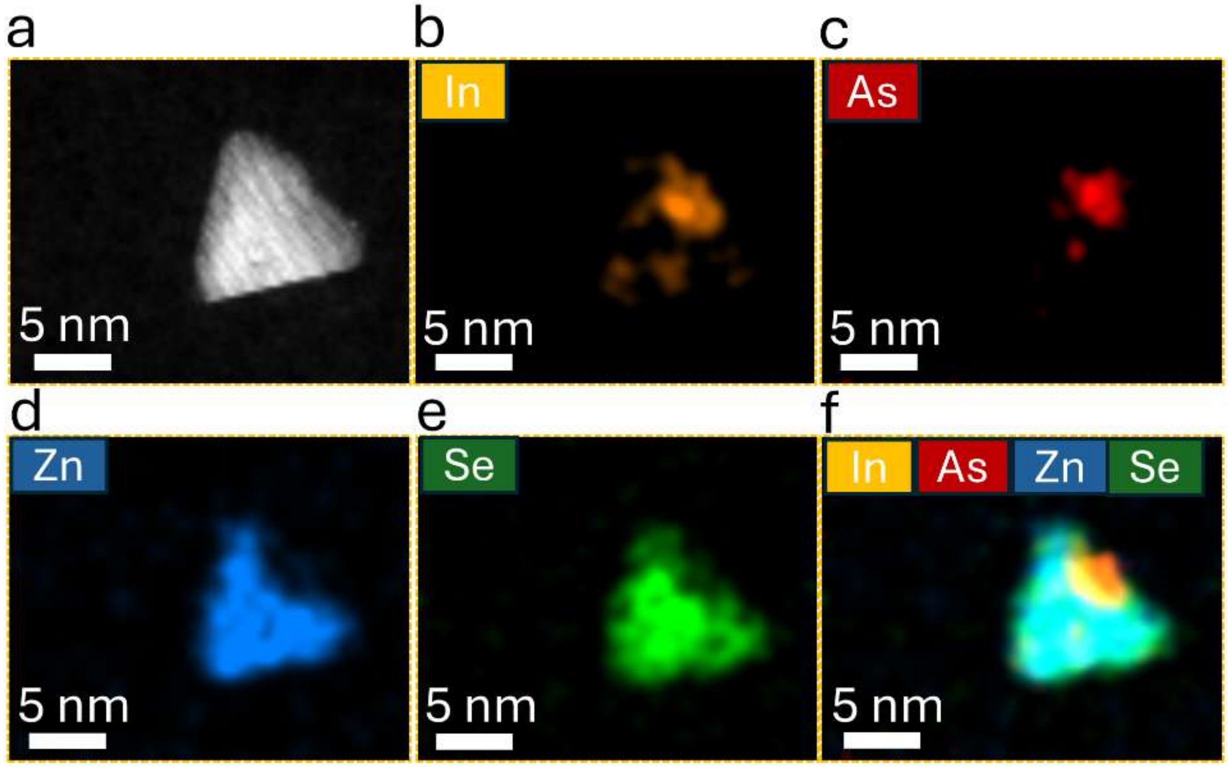


**Figure S9.** EDX elemental mapping of the resulting QD-3 core@shell NCs. The scale bar is 5 nm.

**Synthesis of InAs core NCs with other Grignard reagents**

In addition to BnMgCl, we also carried out InAs core NCs syntheses by employing a variety of other commercially available Grignard reagents. In a typical procedure, a mixture of 0.2 mmol $InCl_3$, 1 mmol of $ZnCl_2$, and 5 ml of degassed OLAM were kept under vacuum at 120 °C for 1 h. Then, 0.6 mmol of corresponding Grignard reagents were added to the mixture and heated up to 240 °C. At that temperature 0.2 mmol amino-As (in 0.5 ml degassed OLAM) was quickly injected. The reaction temperature was then raised to 300 °C and maintained for 15 minutes to allow for crystal growth. Washing of the InAs NCs was performed by adding anhydrous toluene and ethanol to the mixture, followed by precipitation by centrifugation at 6000 rpm for 3 min.

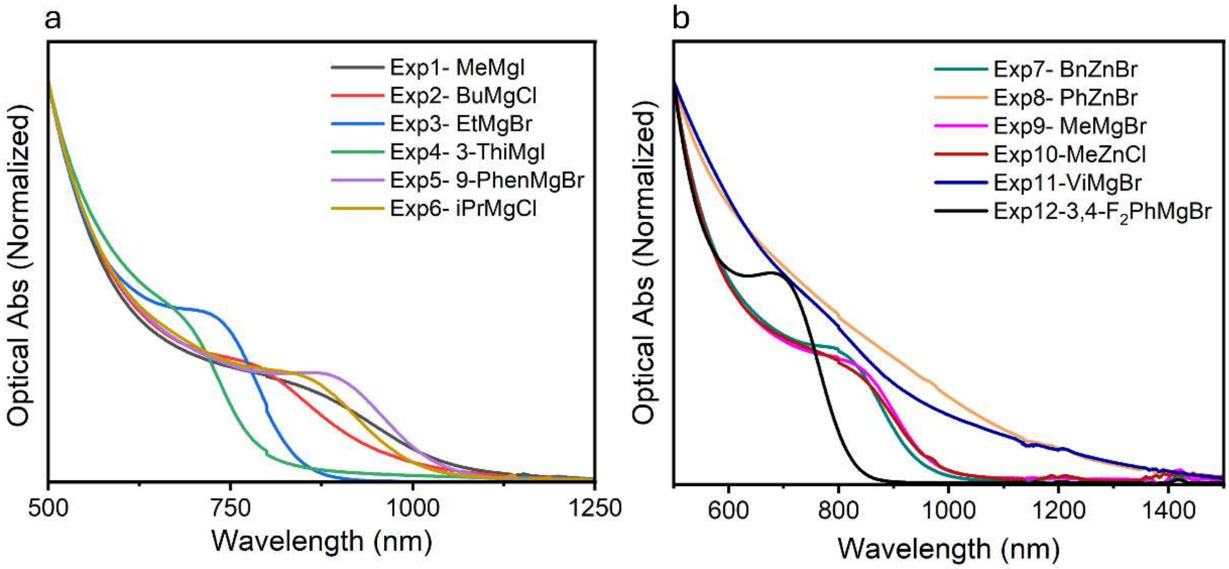


**Figure S10. (a)-(b)** Optical absorption spectra of as-synthesized InAs core NCs using various Grignard reagents with Zn:In ratio of 5:1. For each experimental studies, injection temperatures of Grignard reagent and amino-As solutions were adjusted to 120 °C and 240 °C, respectively. After the injection, the crude solution was heated up to 300 °C for 15 min.

**Table S6.** Grignard reagents used in InAs NC synthesis and their corresponding optical absorption and HWHM values.

| Experiment | Grignard Reagent | Acronyms | Abs (nm) | HWHM (meV) |
|---|---|---|---|---|
| 1 | Methyl Mg Iodide | MeMgI | 870 | >250 |
| 2 | Butyl Mg Chloride | BuMgCl | 790 | 230 |
| 3 | Ethyl Mg Bromide | EtMgBr | 720 | 213 |
| 4 | 3-Thienyl Mg Iodide | 3-ThiMgI | 690 | >250 |
| 5 | 9-Phenanthryl Mg Bromide | 9-PhenMgBr | 875 | 178 |
| 6 | Isopropyl Mg Chloride | iPrMgCl | 840 | 193 |
| 7 | Benzyl Zn Bromide | BnZnBr | 810 | 225 |
| 8 | Phenyl Zn Bromide | PhZnBr | ---- | ---- |
| 9 | Methyl Mg Bromide | MeMgBr | 830 | 236 |
| 10 | Methyl Zn Chloride | MeZnCl | 820 | >250 |
| 11 | Vinyl Mg Bromide | ViMgBr | ---- | ---- |
| 12 | 3,4 Difluorophenyl Mg Bromide | 3,4-$F_2$PhMgBr | 685 | 190 |

**References**


1. Liu, Z.; Pascazio, R.; Goldoni, L.; Maggioni, D.; Zhu, D.; Ivanov, Y. P.; Divitini, G.; Camarelles, J. L.; Jalali, H. B.; Infante, I., Colloidal InAs Tetrapods: Impact of Surfactants on the Shape Control. *Journal of the American Chemical Society* **2023,** *145* (33), 18329-18339.
2. Panda, S.; Zhu, D.; Goldoni, L.; Asaithambi, A.; Brescia, R.; Saleh, G.; De Trizio, L.; Manna, L., Overcoming the Short-Wave Infrared Barrier in the Photoluminescence of Amino-As-Based InAs Quantum Dots. *Advanced Optical Materials* **2025,** *13* (29), e01512.
